# WHF IASC Roadmap on Chagas Disease

**DOI:** 10.5334/gh.484

**Published:** 2020-03-30

**Authors:** Luis Eduardo Echeverría, Rachel Marcus, Gabriel Novick, Sergio Sosa-Estani, Kate Ralston, Ezequiel Jose Zaidel, Colin Forsyth, Antonio Luiz P. RIbeiro, Iván Mendoza, Mariano Luis Falconi, Jorge Mitelman, Carlos A. Morillo, Ana Cristina Pereiro, María Jesús Pinazo, Roberto Salvatella, Felipe Martinez, Pablo Perel, Álvaro Sosa Liprandi, Daniel José Piñeiro, Gustavo Restrepo Molina

**Affiliations:** 1 Department of Cardiology, Cardiovascular Foundation of Colombia, Floridablanca, CO; 2 LASOCHA, Washington DC, US; 3 Medstar Union Memorial Hospital, Baltimore, MD, US; 4 Swiss Medical Group, Buenos Aires, AR; 5 Friedman School of Nutrition Science and Policy, Tufts University, Boston, MA, US; 6 Drugs for Neglected Diseases initiative-Latin America, Rio de Janeiro, BR; 7 World Heart Federation, Geneva, CH; 8 Sanatorio Güemes, Buenos Aires, AR; 9 Pharmacology Department, School of Medicine, University of Buenos Aires, Buenos Aires, AR; 10 Internal Medicine Department, School of Medicine, Federal University of Minas Gerais (UFMG), Belo Horizonte, BR; 11 Hospital das Clínicas, UFMG, Belo Horizonte, BR; 12 Central University of Venezuela, Caracas, VE; 13 Cardiology Division, Italian Hospital of Buenos Aires, Buenos Aires, AR; 14 University Institute of the Italian Hospital of Buenos Aires, Buenos Aires, AR; 15 Faculty of Medicine, University of Buenos Aires, Buenos Aires, AR; 16 School of Medicine, Barcélo University, Buenos Aires, AR; 17 Department of Cardiac Sciences, Cumming School of Medicine Division of Cardiology, Libin Cardiovascular Institute, University of Calgary, Calgary, CA; 18 Southeastern Alberta Region, Alberta Health Services, Foothills Medical Centre, CA; 19 Mundo Sano Foundation, Buenos Aires, AR; 20 ISGlobal, Hospital Clínic, University of Barcelona, Barcelona, ES; 21 PAHO/WHO, Montevideo, UY; 22 National University of Cordoba, Cordoba, AR; 23 DAMIC Institute/Rusculleda Foundation, Cordoba, AR; 24 Centre for Global Chronic Conditions, London School of Hygiene and Tropical Medicine, London, GB; 25 Medical School of Cardiology, University of Buenos Aires, Buenos Aires, AR; 26 Faculty of Medicine, University of Buenos Aires, Buenos Aires, AR; 27 School of Medicine, CES University, Medellín, CO

**Keywords:** Chagas disease, cardiomyopathy, neglected tropical disease, heart failure

## Abstract

**Background:**

Chagas Disease is a neglected tropical disease caused by the protozoan *Trypanosoma cruzi*, with some of the most serious manifestations affecting the cardiovascular system. It is a chronic, stigmatizing condition, closely associated with poverty and affecting close to 6 million people globally. Although historically the disease was limited to endemic areas of Latin America recent years have seen an increasing global spread. In addition to the morbidity and mortality associated with the disease, the social and economic burdens on individuals and society are substantial. Often called the ‘silent killer’, Chagas disease is characterized by a long, asymptomatic phase in affected individuals. Approximately 30% then go on develop chronic Chagas cardiomyopathy and other serious cardiac complications such as stroke, rhythm disturbances and severe heart failure.

**Methods:**

In a collaboration of the World Hearth Federation (WHF) and the Inter-American Society of Cardiology (IASC) a writing group consisting of 20 diverse experts on Chagas disease (CD) was convened. The group provided up to date expert knowledge based on their area of expertise. An extensive review of the literature describing obstacles to diagnosis and treatment of CD along with proposed solutions was conducted. A survey was sent to all WHF Members and, using snowball sampling to widen the consultation, to a variety of health care professionals working in the CD global health community. The results were analyzed, open comments were reviewed and consolidated, and the findings were incorporated into this document, thus ensuring a consensus representation.

**Results:**

The WHF IASC Roadmap on Chagas Disease offers a comprehensive summary of current knowledge on prevention, diagnosis and management of the disease. In providing an analysis of ‘roadblocks’ in access to comprehensive care for Chagas disease patients, the document serves as a framework from which strategies for implementation such as national plans can be formulated. Several dimensions are considered in the analysis: healthcare system capabilities, governance, financing, community awareness and advocacy.

**Conclusion:**

The WHF IASC Roadmap proposes strategies and evidence-based solutions for healthcare professionals, health authorities and governments to help overcome the barriers to comprehensive care for Chagas disease patients. This roadmap describes an ideal patient care pathway, and explores the roadblocks along the way, offering potential solutions based on available research and examples in practice. It represents a call to action to decision-makers and health care professionals to step up efforts to eradicate Chagas disease.

## Introduction

In 2014, the World Heart Federation (WHF) launched an initiative to develop a series of Roadmaps, which identify potential roadblocks on the pathway to effective prevention, detection, and management of cardiovascular disease (CVD), along with evidence-based solutions to overcome them. The resulting documents act as a tool to help turn strategic intent into action plans for integrating the latest knowledge and evidence into national plans for optimal management of cardiac diseases.

The Roadmap publications have become the cornerstone of WHF activities as implementation resources, and they guide initiatives to support heart health globally, translating science into policy and influencing agencies, governments, and policymakers alike. They aim to provide a framework for countries that wish to develop or update national initiatives and programmes tackling cardiac diseases.

Despite more than a century having elapsed since the discovery of Chagas disease Echeverría et al., it remains a major public health concern with significant social and economic burdens in both Latin America and increasingly on a global scale. CD, like other neglected tropical diseases (NTD), is a chronic, stigmatizing condition, closely associated with poverty. Despite being infectious in origin, the predominant and most serious chronic manifestations of CD affect the cardiovascular system. In keeping with WHFs mission to deliver cardiac health for all as a fundamental human right and crucial element of global health justice, we have made the elimination of this disease one of our priorities.

**Figure 1:**
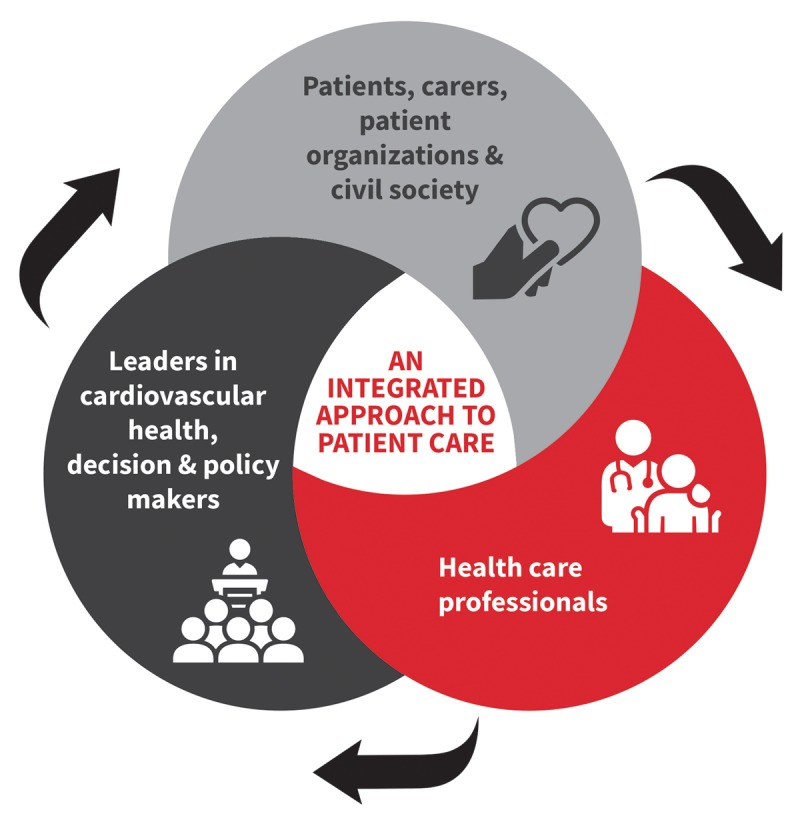
An integrated approach to patient care.

The WHF Roadmap on CD is a document created for all stakeholders to provide an integrated approach to patient care (Figure 1). Its goal is to present a framework for prevention and control efforts at a national, regional, and global level that balances the feasibility, acceptance, and accessibility of solutions presented for local implementation.

**Figure 2:**
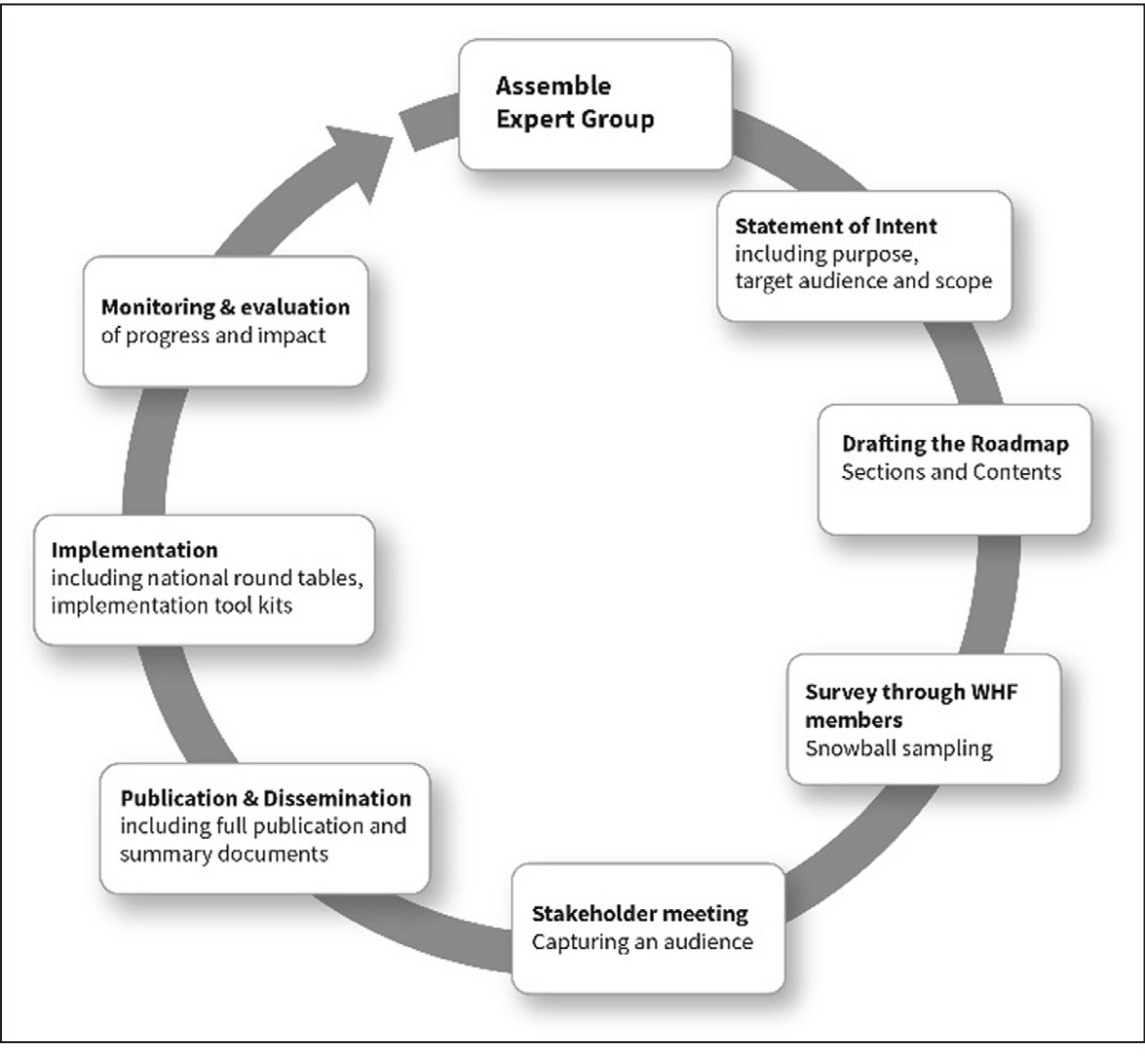
WHF Roadmaps design and methodology.

The Roadmap on CD sheds light on the barriers to patients accessing care, and proposes evidence-based practical solutions for healthcare professionals, health authorities and governments to help overcome these barriers. As part of the WHF Roadmap series, it complements existing Roadmaps on rheumatic heart disease [1], tobacco control [2], hypertension [3], the use of secondary prevention for CVD [4], atrial fibrillation [5], heart failure, and prevention of cardiovascular disease among people living with diabetes [6]. Figure 2 outlines the design and methodology of the WHF Roadmap series.

### Expert writing group

In 2019, the WHF and the Inter-American Society of Cardiology (IASC) convened a Roadmap writing group in joint partnership, consisting of 20 diverse CD experts: clinicians, allied health professionals, health systems experts, and researchers representing many of the important stakeholders working on this disease. Considering CD not only as an infectious disease but as a chronic cardiac disease requiring comprehensive care and management across the lifespan, this Roadmap provides an essential framework for all involved in the planning, organization, patient management and implementation of approaches to CD. It includes an integrated approach from a broad group of stakeholders including healthcare professionals, patients, academic and research institutions, and policy-makers. Recommendations are identified from the standpoint of CD experts as well as those living with CD.

### Methodology

To ensure a best practice approach and a consensus document, the ‘WHF IASC Chagas Disease Roadmap’ was developed through the review of published guidelines and research papers. An extensive review of the literature describing obstacles to diagnosis and treatment of CD along with proposed solutions was conducted. A survey was sent to all WHF Members and, using snowball sampling to widen the consultation, to a variety of health care professionals working in the CD global health community. The results were analyzed, open comments were reviewed and consolidated, and the findings were incorporated into this document, thus ensuring a consensus representation (Figure 2). The ‘WHF IASC Chagas Disease Roadmap’ can be used as a springboard to initiate a *call for action* and prescribe measurable steps towards a common goal, at national and international levels.

### What is Chagas Disease?

CD is a multi-systemic disorder that can affect the cardiovascular, digestive and central nervous systems [7]. CD is caused by *Trypanosoma cruzi*, a hemoflagellate parasite that is transmitted through various species of hematophagous reduviid insects (‘kissing bugs’) whose habitat ranges from Argentina and Chile to the southern half of the United States. The parasite can also be transmitted transplacentally, as well as through infected blood transfusions or organ donations, laboratory accidents, needle sharing among intravenous drug users (IVDU), and orally through food and drink contaminated with triatomines or their feces.

CD is endemic to all continental Latin American countries. The most recent estimates from WHO (2015) indicate a prevalence of 5.7 million in endemic countries, mostly concentrated in Argentina (1,505,235 cases, prevalence 3.6%), Brazil (1,156,821, 0.6%), Mexico (876,458, 0.7%) Bolivia (607,186, the highest prevalence, 6.1%), Colombia (437,960, 0.9%) and Venezuela (310,000, 1.1%) [8].

An estimated 10–14,000 patients with Chagas die each year, and given its poor prognosis, chronic Chagas cardiomyopathy (CCC) is associated with substantial morbidity that correlates with an increased economic burden for individuals and communities [9,10]. Because CD often manifests 15–30 years after childhood infection [7], the impact on earning potential for affected individuals is high, which is catastrophic for socially disadvantaged individuals and their families, among whom CD is disproportionately concentrated.

**Figure 3:**
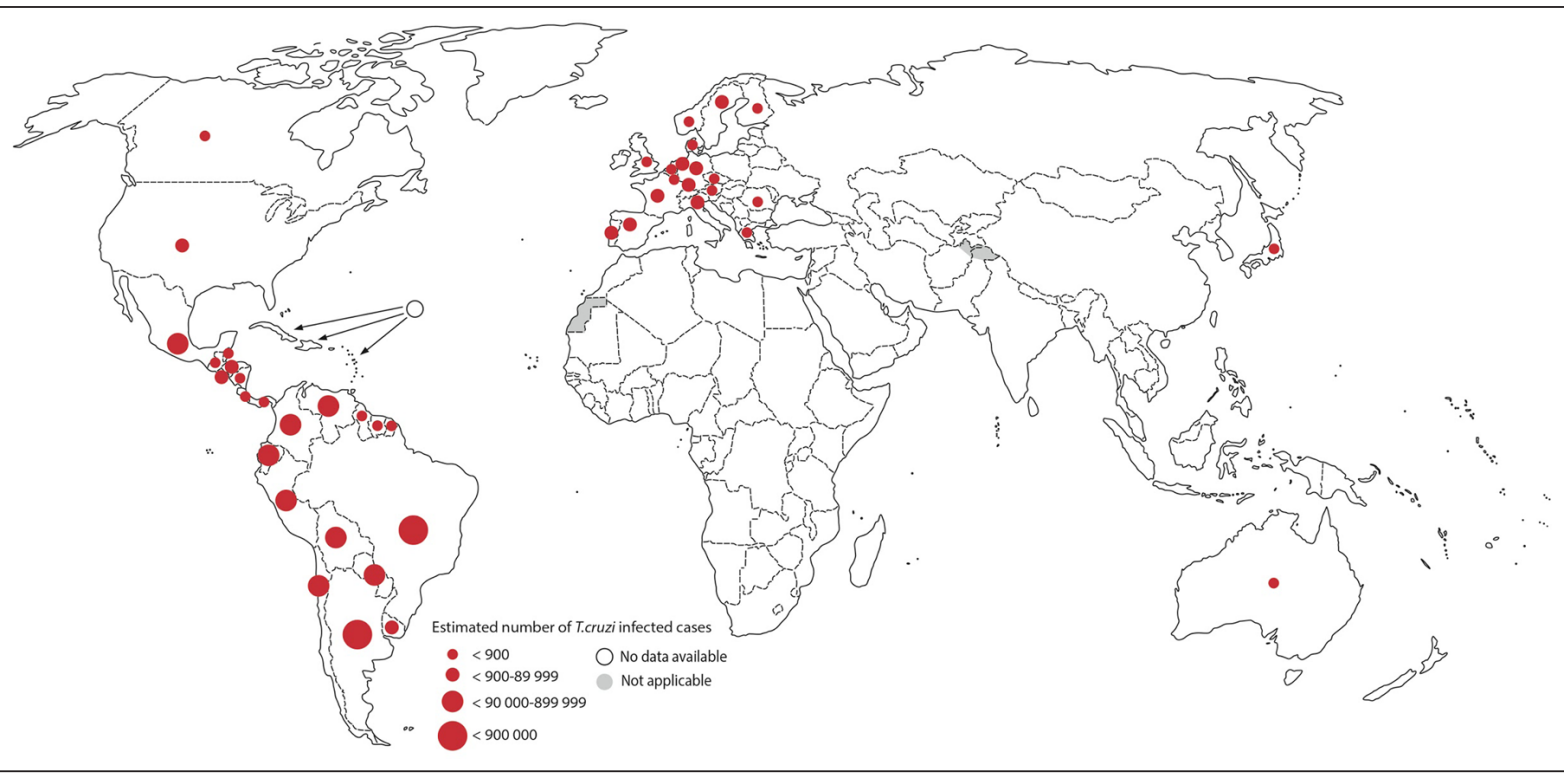
Global distribution of Chagas disease cases, based on official estimates, 2006–2015 [9].

Estimates of CD prevalence have decreased markedly in the last decades, from 17 million in 1980 to less than 6 million in 2010, which has been attributed to coordinated programmes aiming to interrupt transmission of CD [11]. Indeed, as vector and blood transfusion transmission has been interrupted in many countries (Uruguay, Chile, Brazil, Paraguay, Honduras, Nicaragua, Belize, and some states of Argentina, Bolivia, Peru, Colombia, El Salvador, Guatemala, Panamá, Costa Rica), the number of new cases has dropped substantially [12]. However, there are still some areas where vector-based transmission occurs, possibly related to the existence of high-density vector infestation and delayed implementation of vector-control interventions. Most of these new vector-transmitted cases have been reported in Bolivia and the Gran Chaco region, as well as in Central American countries such as Guatemala and El Salvador [8]. Figure 3 shows the global distribution of CD cases, based on official estimates, 2006–2015.

In parallel to this trend of decreasing incidence, several additional phenomena have impacted the changing epidemiological profile of CD over the last two decades:

**Globalization:** Because of political and economic pressures, large numbers of infected individuals have migrated from endemic countries to non-endemic areas, including Europe, Japan, Australia, and the United States. CD has thus become a global health concern. It is estimated that more than 300,000 people with CD live in the USA [13], and another 42,000 reside in Spain [14]. In these countries, physicians are generally unaware of the disease and therefore do not recognize (Figure 3) or treat it, and health system responses to care for affected people have been slow to materialize. In addition, the frequently tenuous social and economic conditions of migrants complicate access to care.

**Aging:** In most places where vector transmission has been interrupted, the majority of individuals with CD are now adults or older persons, and CCC often co-exists with other cardiac risk factors and diseases such as diabetes, coronary artery disease and hypertensive cardiomyopathy [15]. In Brazil, the burden of morbidity and mortality from CD is highest among males, the elderly, and in those Brazilian states encompassing important endemic areas for vector transmission in the past [16]. The natural history of CD in the elderly has not been completely described, although we know that Chagas cardiomyopathy remains a strong predictor of higher risk of death [15].

**Congenital transmission**: While other routes of transmission are decreasing in importance due to successful interventions interrupting vector and blood transfusion routes, congenital transmission has become proportionately more relevant. It now accounts for about one-third of new infections [8], representing the major mode of transmission in non-endemic areas.

**Oral transmission**: Oral transmission is now more frequently recognized, especially in the Amazon region and the subtropical Andes (Venezuela, Colombia and Ecuador) [17]. It is now the primary mechanism of acute cases in the Brazilian Amazon and Venezuela [18,19], and is characterized by higher mortality during the acute phase than the vector-mediated acute disease [20].

**Underreporting**: Most estimates of the burden of CD are based on official mortality figures. However, since CD is a neglected disease that is often not recognized by physicians, it is not included as the cause of death in many cases [21], leading to marked under-recognition of the disease burden [22].

### Natural History of the Disease

**Figure 4:**
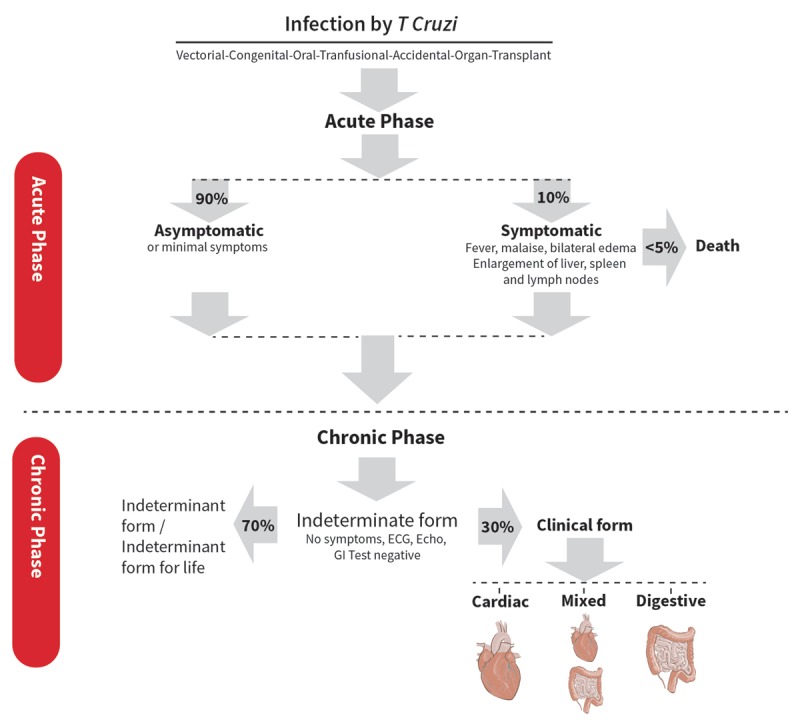
Phases of Chagas disease.

Most CD patients remain asymptomatic throughout life. As shown in Figure 4, approximately 30% progress to clinical forms of the disease, often after a silent phase of many years, and can go on to develop severe clinical complications, mainly cardiovascular, that may lead to incapacity and death [23].

Following an incubation period that varies from seven to fifteen days for vector-based transmission and around thirty to forty days in the case of transfusion-related transmission, the infection passes through two distinct phases. The initial acute phase is characterized by high levels of parasitemia, in which the parasite assumes a trypomastigote form and invades the liver, gut, spleen, lymphatic ganglia, central nervous system, skeletal and cardiac muscles. After the acute phase, *T. cruzi* assumes the dividing form (amastigote), unleashing a local inflammatory reaction [8]. The acute phase generally lasts for one to two months and is followed by an asymptomatic indeterminate phase, during which time no clinical manifestations are observed. In the third of patients that go on to develop chronic CD, the parasite and the immune response cause damage to end organs (Figure 4) [24].

## Clinical manifestations

### Acute Chagas

It is likely that many cases are asymptomatic, or that acutely infected individuals have nonspecific symptoms that do not prompt them to seek medical care, including: fever, fatigue, rash, anorexia, headache, body aches, diarrhea, and vomiting. Among patients who have a clinical evaluation during this phase, marked parasitemia is noted, and clinical signs include hepatosplenomegaly, generalized or local edema (in limbs or face), and lymphadenopathy. In vector-transmitted CD, some specific signs may be present: inflammation at the inoculation site (inoculation chancre), and Romaña’s sign, a unilateral bi-palpebral painless edema [25,26,27]. Severe acute disease occurs in less than 1–5% of vector-transmitted cases, and may present hemorrhagic manifestations, jaundice, myocarditis, pericardial effusion, tachycardia, arrhythmias, atrioventricular block, and, in a small percentage, meningoencephalitis [28]. Severe acute disease also carries a risk of mortality between 0.2–0.5%. The acute phase of orally transmitted CD is associated with higher risk of a severe presentation, as is also the case in immunosuppressed patients, such as patients taking chemotherapy or those with advanced HIV infection [29]. In the case of vertical transmission, the majority of affected newborns remain asymptomatic; however, at least 10% present with hepatosplenomegaly, sepsis, respiratory failure, low birth weight, or premature delivery [24]. Apart from these specific exceptions, in the majority of cases, symptoms related to the acute phase resolve spontaneously and patients remain chronically infected if untreated. A high index of suspicion for CD is therefore necessary to be able to make an early diagnosis and initiate treatment in order to avoid progression to the chronic stage of the disease, which results in end organ damage.

### Reactivated Chagas disease

Pharmacological immunosuppression or HIV/AIDS, particularly with CD4 counts <200, increases the risk of reactivation in patients with chronic *T. cruzi* infection [30,31]. The overall observed prevalence of reactivation in the absence of prophylactic treatment is 28% in transplant patients and 36–40% in people co-infected with HIV/AIDs [32].

In immunocompromised patients, the most frequent manifestations of acute or reactivated CD are prolonged febrile syndrome and neurological manifestations (meningoencephalitis and/or cerebral granuloma). Also frequent are cardiac manifestations (myocarditis, arrhythmias, and cardiac insufficiency). Dermatologic lesions may be observed in transplant patients, including acute panniculitis in the arms, legs and abdomen [33,34].

### Indeterminate phase

After resolution of the initial acute illness, patients generally pass into a phase of CD in which there are no end organ manifestations of the illness in the setting of positive serology. While this often asymptomatic stage persists for most infected patients, some will pass on to the chronic stage of the disease.

### Chronic Chagas disease

#### Cardiac manifestations (chronic Chagas cardiomyopathy)

**Figure 5:**
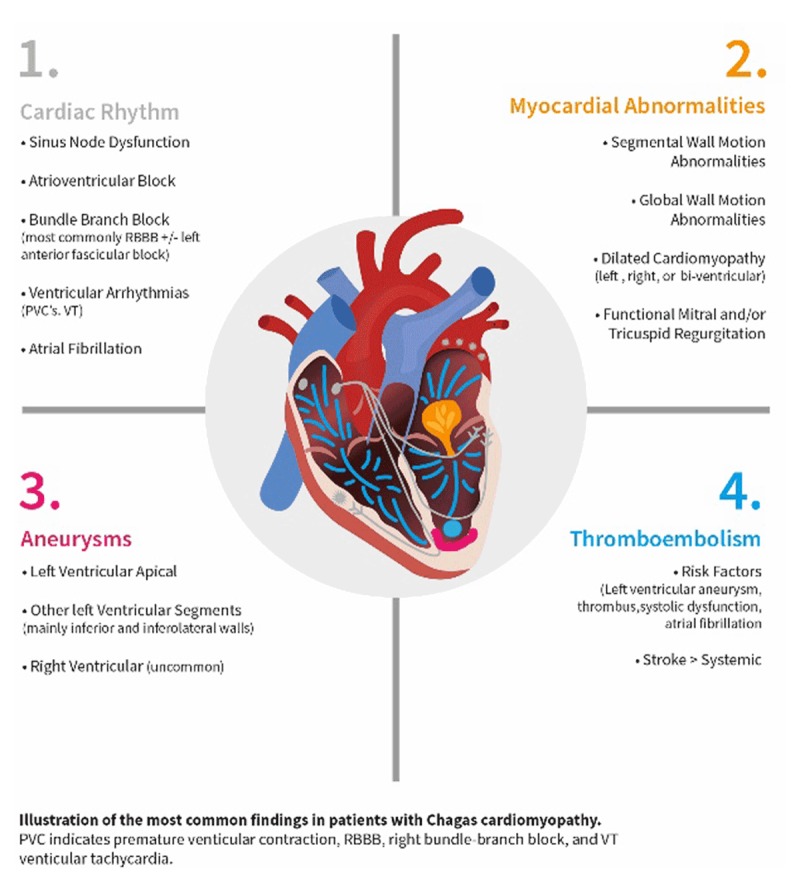
Most common findings in patients with Chagas cardiomyopathy [23].

The 30% of infected patients who progress from the indeterminate phase of the disease develop manifest damage to organs, particularly the heart or viscera [23]. Patients may suffer sudden cardiac death, thromboembolic phenomena, syncope, and congestive heart failure (CHF). Signs and symptoms of cardiac involvement primarily include electrical and mechanical alterations; sinus bradycardia, atrial and ventricular arrhythmias; atrioventricular and intraventricular conduction disorders, such as right bundle-branch block and/or left anterior fascicular block [35]; and ST-T changes. Cardiac imaging demonstrates regional wall-motion abnormalities, apical aneurysms, mural thrombi with embolic potential, and dilated cardiomyopathy with reduced LVEF [36] (Figure 5).

The extent of cardiac involvement in the chronic phase of the disease appears to be the result of the parasite-activated immune response, but parasite persistence during the chronic stage of infection is critical. The immune response elicited in the acute phase and maintained during the chronic one seems to be influenced by variables such as parasite load during the acute phase, parasite strain, the magnitude of the immune response, and the presence or absence of reinfection [7].

CCC has a worse prognosis than other etiologies, with about 10% of patients progressing to terminal CHF, and is also associated with higher rates of hospital readmissions and mortality, regardless of age and in the absence of other comorbidities [7,37,38,39,40].

Cardiac mortality among CCC patients is mainly due to the high prevalence of life-threatening ventricular arrhythmias, manifesting as cardiac arrest and sudden death [23]. Additionally, the association of atrial fibrillation and apical aneurysms, along with a hypercoagulable state from *T. cruzi* infection provokes higher rates of embolic events compared to other heart failure etiologies [35,36].

Although different clinical scoring systems, imaging modalities (Echo, MRI), and several biomarkers including NT-proBNP and Hs-cTnT have been associated with CCC stages of severity and subsequent mortality, the development of better predictors of disease progression and prognosis is still needed [41].

#### Gastrointestinal manifestations

Some patients, especially those infected with strains of the parasite found in the southern countries of Latin America (Brazil, Bolivia, Argentina) can present with the digestive form of the disease. This form, which involves denervation of the autonomic plexuses of the digestive tract, leads to disturbances in absorption, motility, and secretion. Motor incoordination and subsequent dilatation result in megaviscera, involving mainly the esophagus and the colon [7]. Symptoms of megaesophagus include those typical for achalasia, such as dysphagia (retention of food in the esophagus), and symptoms of megacolon include constipation, often profound, with rare volvulus requiring surgical correction.

#### Central nervous system involvement in chronic CD

In chronic CD, central nervous system (CNS) involvement is rare but associated with a poor prognosis. Most cases of CNS involvement are due to CD reactivation, especially in immuno-suppressed patients.

## Patient pathway of care

**Figure 6:**
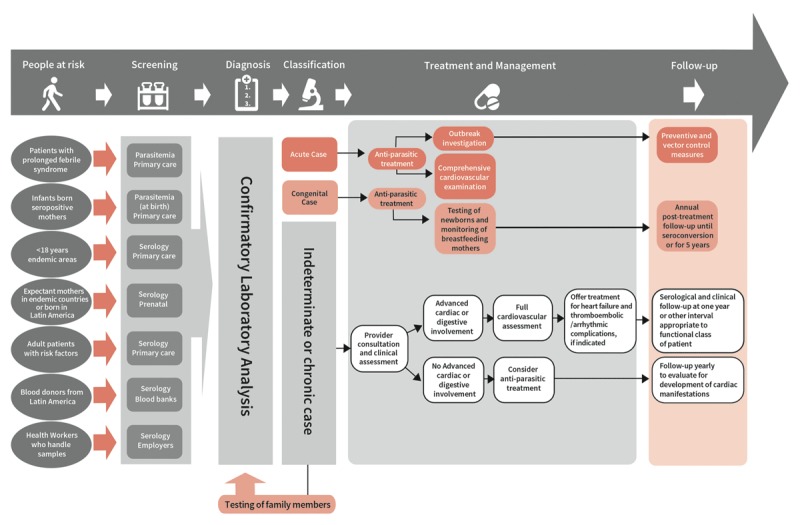
Ideal patient care pathway, Chagas disease.

Figure 6 shows an ideal patient journey for individuals affected by Chagas disease and represents a minimal standard for comprehensive care through all levels of interventions. In the following sections of the document, as we move along the steps of the journey and levels of intervention, we will endeavour to highlight gaps and roadblocks in the current standard of care, make recommendations and propose evidence-based solutions.

## Prevention of Chagas Disease: Primary, secondary and tertiary interventions

## Primary prevention

### Preventing vector-based transmission

**Figure 7:**
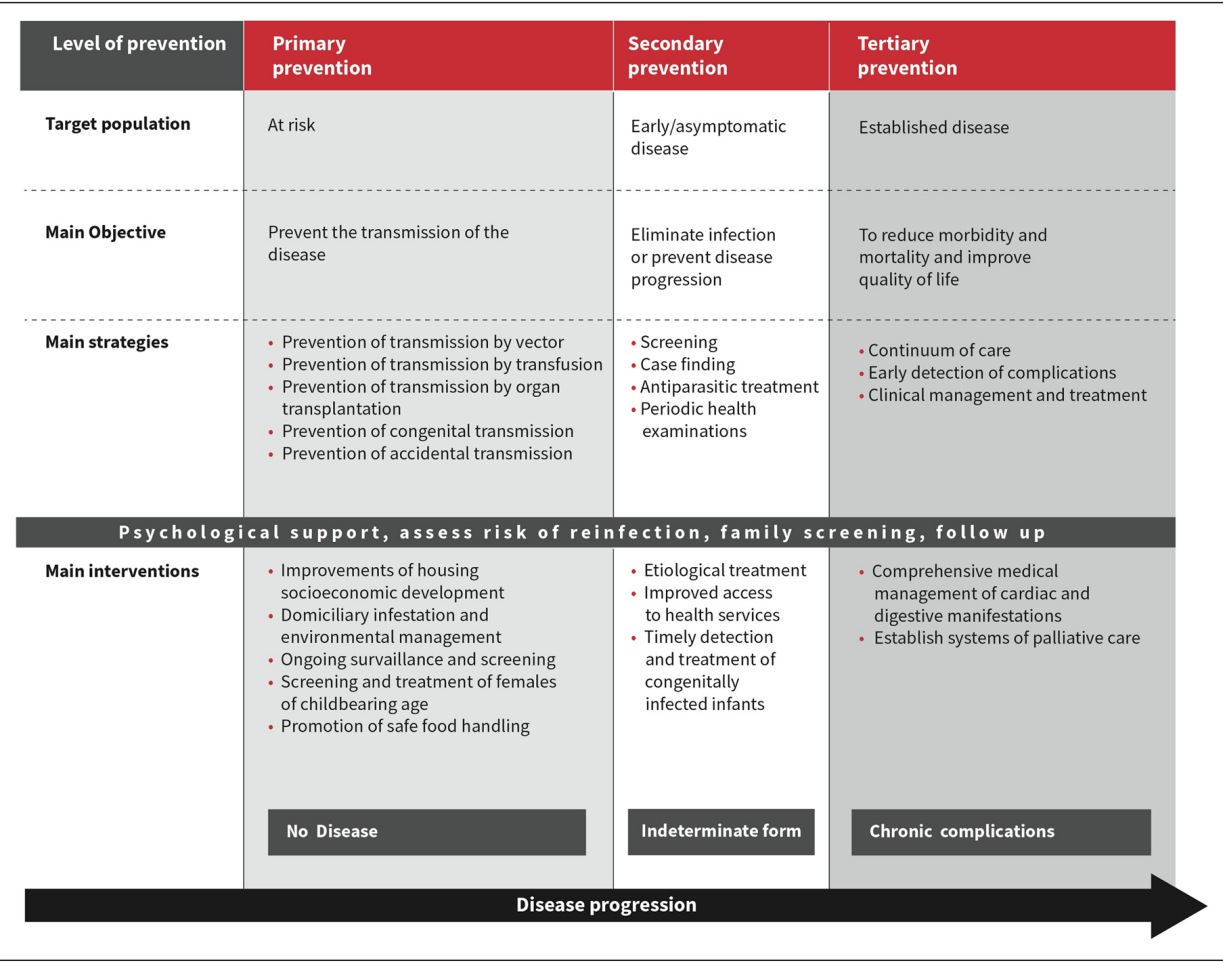
Levels of prevention and main interventions.

Vector control measures have achieved substantial success at eliminating transmission by domiciliary vectors in several countries, and as a result have been highly effective at reducing new vector-mediated cases of CD (Figure 7). Nevertheless, there are some remaining foci, notably in Central America and the Chaco region, where vector-based transmission persists. Reasons for this include reduced efficacy of pesticides in houses made of natural materials, and cases of pesticide resistance [42,43]. The elimination of sylvatic vectors is virtually impossible and in some cases, they may occupy the niches left vacant by the elimination of domiciliary vectors. The re-emergence of vectors is a potential issue in areas where surveillance is weak and spraying programmes are sporadically enforced. In addition to this, political commitment to maintaining surveillance may wane once an area is certified as free of transmission [44]. Vector-based transmission of *T. cruzi* is closely associated with socioeconomic conditions, and is strongly associated with housing constructed of natural materials (mud, adobe, thatch).

The most sustainable way to interrupt vector-based transmission in remaining foci of infestation is through housing improvement and community development programmes that are supplemented by regular, systematic pesticide applications [43]. Successful control strategies will address both domestic and peridomestic structures and raise awareness in the affected communities about the reasons for these efforts [43,45,46]. Strengthening of vector surveillance in endemic areas to allow for timely reporting of infestations, ideally as a collaborative effort between communities, health systems, and vector control teams, can help prevent re-emergence of vector-based transmission in areas where this modality of transmission has been interrupted.

### Preventing congenital transmission

Congenital transmission is most effectively interrupted by screening all women of childbearing age and treating seropositive women with anti-parasitic agents prior to pregnancy. Several observational studies indicate that anti-trypanosomal treatment of women is dramatically effective in preventing congenital transmission in future pregnancies [47,48,49]. While contraindicated during pregnancy, treatment of females of childbearing age is now recommended in major guidelines [50,51]. Although new national and regional initiatives aim at reducing congenital transmission, many gaps remain. Healthcare personnel are often unfamiliar with the risk of congenital transmission [52]. Screening of pregnant women is far from universal, and totally absent in some affected countries [53,54,55], despite the demonstrated cost-effectiveness of these programmes in both endemic and non-endemic settings [53,55,56]. The regional initiative Eliminating Mother to Child Transmission Plus, launched by PAHO in 2017 [57], works to strengthen health systems in participating countries to interrupt congenital transmission of CD along with other congenitally transmitted infectious diseases, although the recommendations in this document are far from being widely adopted in the region.

For pregnant women, a universal serological screening protocol during prenatal visits should be established at the primary care level and integrated into existing perinatal care structures. Clear guidelines and procedures for verified cases of infected newborns should be developed, including serological monitoring for infection of any babies born to infected mothers who do not have overt parasitemia at birth. Decentralized models of care, such as outreach primary care should also include local medical facilities for serologic testing and clinical follow-up of infected adults, children and pregnant mothers, in a timely manner.

### Preventing oral transmission

Orally acquired CD, an important syndrome in the Amazon region, is associated with a potentially virulent acute phase [20]. Because oral transmission occurs primarily through food, especially fruit contaminated with triatomine feces, implementation of safe food-handling practices combined with health promotion activities on the prevention of oral transmission are needed to prevent continued oral CD outbreaks. Promotion of safe food-handling practices among households and food vendors in areas at risk is essential, especially for high-risk foods such as sugar cane and acai [58,59].

### Preventing transmission via blood transfusion, organ transplant, and laboratory accidents

There has been growing acceptance of the need for screening of blood donations, in both endemic and non-endemic countries over the past 30 years, resulting in a significant decrease in known transfusion-related diseases. Nevertheless, screening remains only partially implemented and is essential for preventing transfusion and transplant transmission [60,61,62]. Prevention of accidental transmission in laboratory settings is best achieved through rigorous maintenance of safe laboratory practices in those institutions where *T. cruzi* and/or infected triatomines or blood and tissue samples are handled [63,64,65].

CD blood bank testing protocols should be in place with specific technical guidelines, collection protocols, reporting, and counselling and follow-up of positive cases. Special attention should be given to confirming inconclusive results with a combination of tests, given that the estimated prevalence of CD has been reported to be 13.30% in the stratum of donors with inconclusive serology at screening, and up to two false-negative results have also been reported, depending on the technique used for testing [32,66].

**Roadblocks to primary prevention:**

- Discontinuation and interruptions of vector control interventions threaten to stall remarkable progress towards the elimination of vector-transmitted disease.
- There is inadequate focus on continued surveillance and eradication.
- Ongoing congenital transmission is facilitated by the lack of widespread adoption of screening recommendations to achieve early diagnosis and treatment.

## Secondary prevention: Diagnosis and treatment

Timely diagnosis and treatment is critical in order to improve treatment success rates and prevent progression to the chronic phase of the disease. As part of a comprehensive approach, the diagnosis and treatment process should involve screening for chronic manifestations of the disease at the point of care. A thorough clinical review, including medical history and physical examination, should seek to elicit clinical signs and symptoms not only of acute CD but also cardiac and gastrointestinal chronic manifestations. As a minimum, an ECG should be performed to detect cardiac involvement in all patients with positive serology.

In order to vastly improve currently inadequate diagnosis rates, all individuals from endemic areas should be screened for CD on at least one occasion. In endemic regions, pregnant women as well as newborns from infected mothers should be tested. Blood donors should always be tested.

In non-endemic settings where screening is not universally offered, healthcare providers should consider the following risk factors when determining who to test:

– Having lived in an endemic area.
– Having lived in a rural area of an endemic country, particularly in housing made of natural materials (mud, adobe, thatch, palm leaves).
– Having a family member with CD, especially a sibling with congenital CD.
– Having been born to an infected mother (if the answer is no, it is important to ask for the epidemiological background of grandmothers and other family members, as well as a history of cardiac disease).
– Having been in contact with kissing bugs, particularly having seen them in the home or recalling bites.
– Having received blood transfusions, or having undergone major surgery (during which a blood transfusion may have occurred).
– Having received organ transplants in endemic areas.
– Having a history of intravenous drug use in an endemic country.

Immunocompromised patients with *T. cruzi* infection must be under clinical and laboratory monitoring for acute reactivation. Any clinical signs such as fever, neurological findings or acute myocarditis must be considered as warning signs of infection reactivation [67].

### Diagnosis of *T. cruzi* infection in the general population

In areas with endemic vector-borne transmission, acute CD is generally a disease of childhood, although there are notable exceptions such as orally transmitted infection. Given the non-specific clinical manifestations, the diagnosis of acute CD is rarely made. However, if suspected, detection of the parasite via direct observation in the blood and/or molecular diagnosis are the first line techniques due to the high level of parasitemia in this phase. Serological testing is the second option, since antibodies may not yet be detectable.

**Figure 8:**
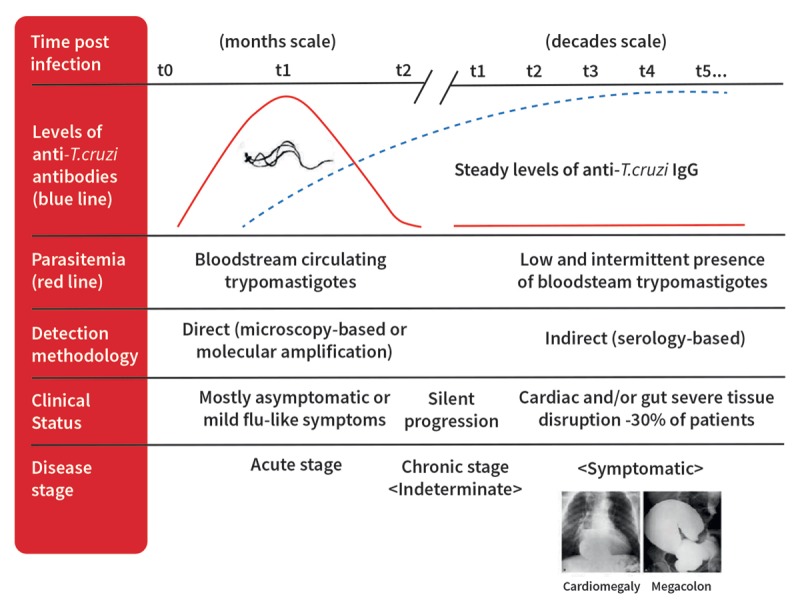
Timescale of Chagas disease diagnosis and clinical status progression [68].

Regardless of the mode of transmission, without treatment CD exhibits a trend of parasitemia that peaks around 30 days after infection (Figure 8). It then declines steadily until around 90 days after infection, when the parasite becomes undetectable by direct microscopy and specific antibodies against *T. cruzi* are present in blood.

During the chronic phase of the infection, which is lifelong in the absence of successful antitrypanosomal treatment, parasitemia remains below microscopically detectable levels, and diagnosis relies on serologic assays to demonstrate IgG antibodies against the parasite. To date, no single assay is sufficiently sensitive and specific to define infection. Positive results by the screening assay must be confirmed by a second test; hence, the diagnostic process is cumbersome. Additionally, the performance of diagnostic tools is highly variable [69,70]. Simplifying diagnosis with high quality rapid testing that does not require external confirmation is critical to timely diagnosis, ideally utilizing a test that performs acceptably with patients at risk for infection with a variety of Trypanosoma strains.

There are currently a host of other barriers that are severely restricting access to timely and accurate diagnosis for at risk populations [71,72]. The awareness of CD in the population and among healthcare providers is low, and there is a lack of knowledge on who to screen as well as a lack of clarity on the appropriate tests and interpretation of results [73]. There is an underappreciation of the importance of early diagnosis and treatment, especially at the primary healthcare level, and this represents a missed opportunity for treatment in the early phases, when treatment success rates are higher, and before severe organ damage occurs.

### Diagnosis of *T. cruzi* in special populations

Diagnosis in newborns is made by direct visualization of trypomastigotes in the blood. This method is highly specific and confirms congenital infection, but it has low sensitivity that can be influenced by low parasitemia or an inexperienced technician [74]. Usually, at least, two blood samples are needed, one in the perinatal period and the second up to one month after birth. If positive, the newborn should be treated. If negative, a serologic test is required after the tenth month of age, when maternally transferred IgG antibodies have disappeared. In many regions, the required schedule of testing can make follow-up difficult, due to barriers to healthcare as well as migratory flows of populations [55,68,75]. This may account in part for the estimation that one in two of all congenital infections with CD are missed [76]. New molecular diagnostic tools such as PCR, which is used in the United States for the diagnosis of congenital CD, may eventually facilitate less cumbersome diagnosis of such cases if they can be successfully adapted to clinical settings [24,77,78,79,80].

In patients who are immunosuppressed, detection of the parasite by a direct parasitological test or exponential increase of parasitic load on quantitative PCR must be considered a reactivation, and the affected individual should receive anti-parasitic treatment immediately to avoid severe morbidity and/or mortality [81,82].

### Anti-trypanosomal treatment during the acute phase

Anti-parasitic treatment should be provided as soon as possible following detection of acute *T. cruzi* infection. Treatment during the acute phase is highly effective, producing serological cure, reducing potentially severe clinical manifestations of the acute phase, and preventing progression to chronic CD [83,84].

The efficacy of treatment in the acute phase is almost immediately demonstrable by negative parasitemia with direct or indirect parasitological testing. In addition, antibodies disappear completely (sero-negativization) in at least 65% of cases, with some studies demonstrating sero-negativity in almost 100% of cases within 18 months of follow-up after treatment. This effect is independent of patient age. The absence of parasitemia, demonstrated by direct methods such as Strout or micro method, and negative PCR results always precede the reduction of antibodies [85,86,87]. Parasitic negativization occurs in the majority of treated newborns. Long-term follow-up studies are necessary to assess the relationship of treatment to rates of subsequent clinical events in infants [88,89,90,91,92].

### Anti-trypanosomal treatment during the chronic phase

**Figure 9:**
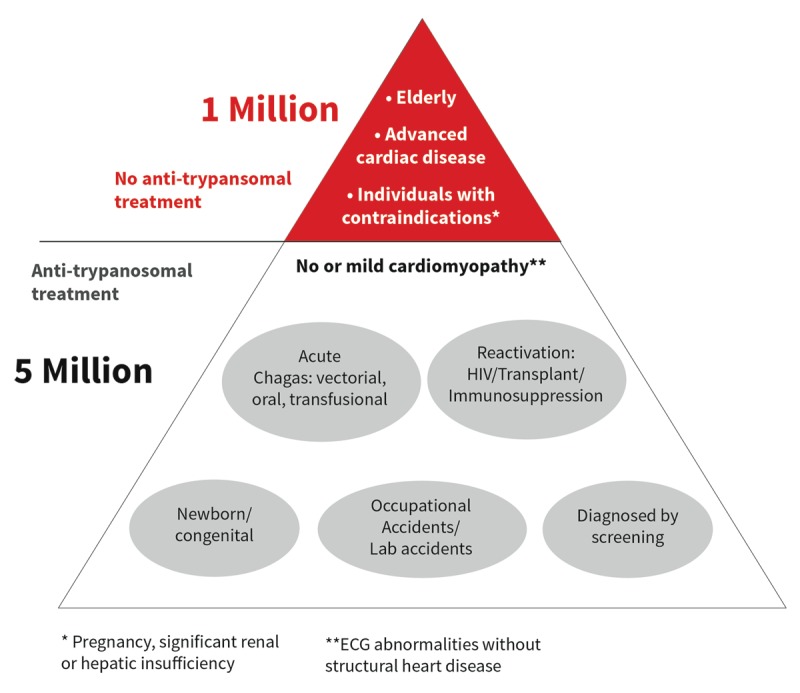
Indications and contraindications for anti-trypanosomal treatment and number of people in both categories globally.

Anti-parasitic treatment during the chronic phase has an acceptable safety profile and is better tolerated in children than adults. However, around 17–35% of adult patients suspend the treatment due to side effects [93,94]. The most frequent and serious are cutaneous, neurologic, hepatic, and hematologic. Anti-parasitic therapy is contraindicated in pregnancy, but may be offered during breastfeeding if indicated [95,96]. Other contraindications include renal or hepatic insufficiency and advanced cardiomyopathy (Figure 9).

**Figure 10:**
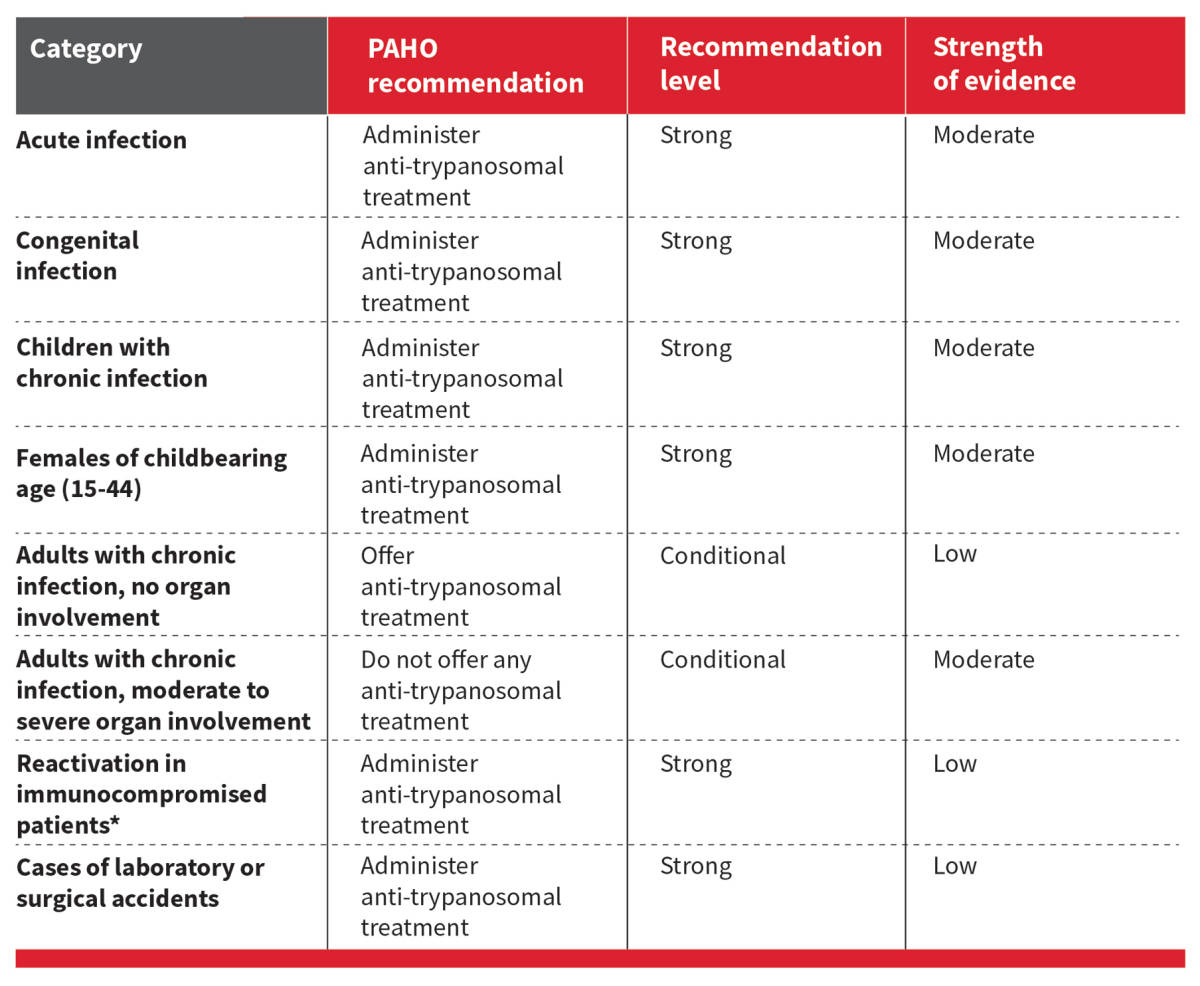
Anti-trypanosomal Treatment Recommendation. Adapted from Guidelines for the diagnosis and treatment of Chagas disease. Washington, D.C.: PAHO; 2019.

In the absence of a gold standard, serological tests to confirm cure and molecular tests to demonstrate failure are the best available tools to assess response to antiparasitic treatment in the chronic phase. While positive PCR during the first 24 months after treatment is indicative of therapeutic failure, sensitivity is variable [97,98]. Post-treatment sero-negativization can take several years, depending on: (i) the age of the individual at time of treatment; (ii) the time elapsed between treatment and follow-up, and (iii) the region where the individual was infected [98,99,100]. Although complete sero-negativization can be obtained within five years in more than 70% of children treated, this rate only reaches about 30% in adult patients after roughly 20 years of follow-up (Figure 10) [48,101,102,103,104].

Although several observational studies show that trypanocidal treatment prevents morbidity and mortality [102,103,104], a randomized control trial of adults with established cardiac disease did not demonstrate clinical benefit after six years of follow-up [93]. In the absence of randomized trial evidence addressing the reduction of clinical events associated with anti-parasitic therapy, the efficacy of anti-parasitic therapy in indeterminate phase adults remains unknown, and demonstrates the need for further research to develop medications with better efficacy in this patient group.

Recent clinical trials indicate a high (≈80%) rate of negativization by PCR in adults undergoing treatment with benznidazole [105,106,107]. In a recently concluded study, this rate was maintained after only two weeks of treatment with the standard dose, and no adverse events occurred [108]. However, new chemical entities (posaconazole and fosravuconazole) did not successfully demonstrate anti-parasitic efficacy in recent research [105,106,107].

Because of the high rate of side effects associated with current anti-parasitic therapy, treatment must be accessible in healthcare facilities near affected communities, and integrated into the local primary care infrastructure. This should be coupled with culturally sensitive educational health promotion programs about the importance of treatment and information on possible side effects. Community-led patient support groups also have an important role to play in providing information regarding CD and treatments. Healthcare staff should be adequately trained to deliver appropriate treatment and manage side effects, in keeping with locally adapted guidelines. Comprehensive follow-up of patients should also involve assisting them in navigating the healthcare system and addressing social or environmental hurdles that could interfere with treatment adherence (Figure 10).

Improved regimens of benznidazole and nifurtimox along with more research into their efficacy in adults, as well as development of new drugs with improved safety profiles and tolerability, are an important future step. However, providers serving affected populations should also receive tools and training to provide optimal treatment with the current drug regimens according to PAHO and national guidelines. Another crucial need is the development of biomarkers to help clinicians assess treatment response. Testing and treatment need to be included in public and private insurance plans, and should be available at no cost to low-income patients in facilities that are near affected communities.

**Key treatment interventions**

**Roadblocks to secondary prevention:**

- Inadequate screening programmes hampering early diagnosis and treatment.
- Inadequate knowledge of the diagnostic process and treatment recommendations in the healthcare community.
- Underappreciation by healthcare professionals of the importance of early diagnosis and treatment, and of screening for early stages of end organ damage.
- Cumbersome diagnostic process, with suboptimal test performance that requires confirmatory testing. Poor availability at the point of care.
- Inadequate investment in R&D for effective anti-parasitic agents with acceptable side effect profiles.
- Prohibitive cost of medical care and other out-of-pocket expenses.

In the words of one patient who spent over 10 years seeking treatment, ‘I thought I had a doctor, a clinic who could help me, but Chagas taught me this is not always so. Because nobody, I mean nobody knew what CD is. My doctor didn’t know, and he sent me to a specialist, and every week I went to a different doctor, but nobody knew what to do with me. And after a year I gave up.’ [109].

## Tertiary prevention

Tertiary prevention involves activities that mitigate or reduce morbidity and mortality from cardiac and other complications caused by CD, and improve the quality of life of affected people. Often, after a long asymptomatic phase, CCC is the most important clinical manifestation of the disease, resulting in the main burden of CD morbidity and mortality [110].

### Clinical management and treatment of chronic Chagas cardiomyopathy

When the acute phase of CD is symptomatic, treatment has two purposes: clinical stabilization of the patient, and control of infection [7]. Management of the clinical consequences of myocarditis caused by *Trypanosoma cruzi* does not differ from other types of infectious myocarditis except for the use of anti-trypanosomal treatment [111]. Approximately 30% of individuals with positive serology without abnormal cardiac function at baseline will eventually develop myocardial dysfunction [23]. Compared to non-infected patients with ventricular dysfunction of other etiologies, CD patients have higher rates of conduction abnormalities, lethal arrhythmia, thromboembolic events, and ultimately mortality [39]. In addition, CCC has a worse prognosis in terms of impact on the quality of life, with higher rates of hospitalizations as a result of these complications [39]. The few studies that have evaluated guideline directed medical therapy in CCC have been compromised by small sample sizes, and most are non-randomized. However, this panel of medical therapy is accepted as appropriate, and includes beta-blockers, angiotensin-converting enzyme inhibitors/angiotensin receptor blockers, angiotensin receptor-neprilysin inhibitors (ARNI), and mineralocorticoid receptor blockers [112]. Because of the unique pathophysiology of CCC, new protocols specifically evaluating therapies in this population are critical, and include PARACHUTE-HF (sacubitril-valsartan) [113] and COACH (colchicine) [114].

Diuretic therapy and digoxin use, while not studied in Chagas cardiomyopathy patients specifically, are appropriate medical therapies to improve functional class and control symptoms in Chagas heart failure. There are theoretical concerns about digoxin effects on an already damaged conduction system, so it should be used with caution. Amiodarone is frequently used to suppress ventricular tachyarrhythmias as well, also in the absence of randomized trial data establishing a benefit. Stroke, systemic, and pulmonary embolism are common in CD, more so than in heart failure of other etiologies. The incidence of ischemic stroke in patients with CCC has been reported to be as high as 2.67 events/100 person-years [115]. Apical aneurysms, found in up to 50% of symptomatic CCC patients, and higher rates of atrial fibrillation in CCC contribute to this elevated thromboembolic risk. A score of at least four points on the well-known Sousa Scale confers a stroke risk of 4.4% per year, which theoretically would overcome the risks of use of warfarin or a direct anticoagulant [116]. Despite the logic of initiating anticoagulation in these individuals, there are still no published randomized clinical trials to confirm the benefit of anticoagulants in CCC in a primary prevention setting, particularly that acknowledge the unusual mechanisms of stroke that this disease confers.

Because of the higher rate of arrhythmia in CCC, electrophysiologic interventions are a cornerstone of therapy for this disease. Amiodarone is widely used, although studies are conflicting in regard to its effect on mortality. Device therapies used in CCC include implantable devices such as implantable cardioverter defibrillators (ICDs) and cardiac resynchronization therapy (CRT). Although use of CRT is strongly supported by trial evidence in heart failure in general, there have been no randomized trials to date that have evaluated these therapies in CCC. Importantly, observational studies have not ubiquitously shown a benefit, underscoring the substantial need for performing studies of both ICDs and CRT in CCC patients, as well as the usefulness of amiodarone versus ICDs in CCC [117,118]. Any study of ICD therapy would ideally address the higher risk among Chagas patients for lethal arrhythmias at ejection fractions that are higher than those that usually confer risk in heart failure of other etiologies.

In the past, CCC has been considered a potential contraindication to transplantation because of concern about reactivation of infection. Nevertheless, in spite of frequent but easily treatable acute reactivation events after transplant, acceptable survival rates have been demonstrated. Because of these results, CCC has not been considered a contraindication for transplantation since the 1990s [119].

### Evaluation and treatment plan for CCC

**Figure 11:**
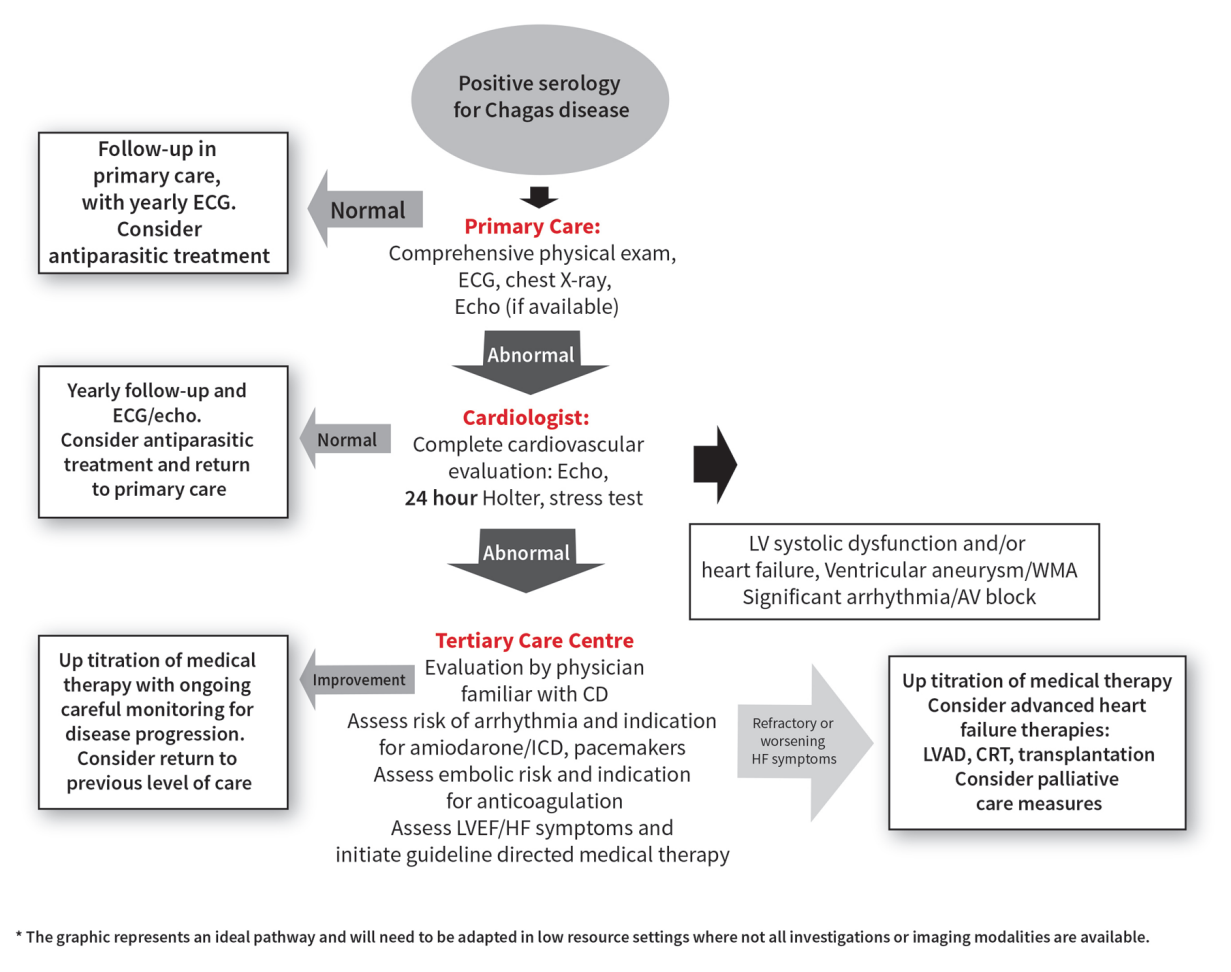
Algorithm for cardiovascular evaluation in patients with positive serology for CD.

Figure 11 delineates the ideal treatment algorithm for a patient with positive serology for CD. Initial cardiac evaluation can be performed at the primary care level, but when ECG changes are present, evaluation by a cardiologist is appropriate. In the presence of significant structural heart disease or arrhythmia, evaluation by a heart failure/cardiomyopathy specialist with experience with Chagas disease is critical, to make sure that appropriate emphasis is placed on risk stratification for stroke and arrhythmia. This is particularly important in non-endemic countries where the disease is not well understood. In the presence of clinical deterioration, patients should be assessed in a tertiary care centre with advanced heart failure treatment options.

Many critical barriers exist to timely, appropriate care for CCC. Low provider awareness, including the frequent under-recognition of CD as the cause of cardiomyopathy, can result in substandard management. Additionally, many imaging modalities, complementary exams and necessary devices (pacemakers, cardioverter-defibrillators) are not available in low-resource settings [120]. The coexistence of advanced chronic CD with a range of other comorbidities, including diabetes, hypertension, depression, or coinfection with HIV, means that CD may be overshadowed by other more ‘urgent’ medical needs [121,122,123].

Clear, rapid referral pathways to tertiary care centres are needed from the primary care level in order to access necessary imaging and investigations, as well as cardiologists and other specialists. Interventions which increase the availability of specialized care to patients in non-urban settings (e.g. telemedicine, mobile clinics) are useful to increase patient access. Ongoing monitoring of patients should be systematically coordinated locally with patients’ primary healthcare service.

In terms of treatment for individuals with moderate to severe cardiomyopathy, R&D for new treatments capable of reversing or negating organ damage secondary to *T. cruzi* infection are urgently needed, thereby acknowledging that Chagas cardiomyopathy is a distinctive and more frequently lethal form of cardiomyopathy.

**Roadblocks to tertiary care:**

- Therapies for advanced disease are costly and frequently only available in urban centres, which are often far from where patients reside.
- Inadequate research has been conducted to evaluate which individuals will progress to clinically significant disease.
- There has not been adequate research to prove that accepted heart failure therapies are effective in CCC, and how to address the higher risk of thromboembolic phenomenon and ventricular arrhythmias in this disease.
- New therapies for advanced CCC that halt disease progression are needed.

## Chagas Disease: Key Issues

### Inadequate R & D

While significant progress has been made in the understanding of CD in the century since Carlos Chagas’ landmark research, there are still only two available anti-trypanosomal drugs, both having unsatisfactory efficacy and safety profiles. Funding for R&D to improve treatments is strikingly low, given the 75 million people at risk in Latin America alone and the significant financial and social burden of the disease. Less than $1 million USD, representing only 0.04% of R&D funding dedicated to neglected diseases, was spent on the development of new drugs for CD in 2007. Although there has been some improvement in the last decade, there is still much work to be done [124,125]. For a disease of global public health importance affecting millions, renewed impetus to develop improved treatments is well overdue. A substantial increase in R&D investment, preferably via product development partnerships (PDPs), will be critical to addressing the need for better pharmacologic therapy. PDPs success in providing patients in resource-poor settings with important therapeutic improvements against malaria demonstrate the efficiency of such collaboration [126].

Although there have been some new developments in R&D from recent clinical trials, there is an urgent need to review and challenge the current evidence and define clinical research priorities for the immediate future. This would ensure that appropriate, efficacious drugs for CD are developed, but will require a focused and collaborative effort from the entire CD research community [127].

Additional anti-trypansomal drugs are currently under development, and there are also CD vaccine (immunotherapeutic) candidates at the pre-clinical stage [128]. In this respect, a PDP is exploring an approach that links therapeutic vaccination to pharmacotherapy [129].

### Scaling up access to diagnostic testing and treatment

The last decades have seen significant changes in approaches to the treatment of CD. Anti-trypanosomal treatments were previously reserved for acute cases and children, but improved knowledge of the pathophysiology of CD and a subsequent shift in thinking has meant that recommendations for testing and treatment now cover a much larger population group, including adults in the indeterminate phase. While randomized trial evidence is not yet available to assess efficacy of anti-parasitic therapy in indeterminate phase adults, recommendations suggest consideration of therapy in this population based on expert consensus. The prioritization of public health resources to focus on vector control programmes as the most cost-effective mechanism to reduce the burden of CD was a rational approach, and continuation of these programmes remains critical to maintaining success. However, it is now time to scale efforts up to provide easier access to diagnostic testing and medical therapy for those with established CD.

Recent PAHO guidelines recommend more widespread treatment of adults in the indeterminate phase of the illness, but to date the ‘treatment gap’ remains vast. With the exception of Spain, non-endemic countries do not have an organized approach to screening or treatment, largely due to a lack of awareness that individuals with CD now reside outside of endemic. Additionally, despite the widespread acceptance of the benefits of prenatal screening, such programmes are not widespread across the Latin American region.

Several examples of local multi-stakeholder initiatives involving national and local governments provide successful models to scale up diagnosis and treatment in the adult population. In collaboration with the Spanish organization IS Global, the Chagas Platform in Bolivia provides an example of scaling up diagnosis and treatment in adults [130]. Under the umbrella of the National Healthcare System’s Chagas National Programme (ChNP), the platform created several specialized centres for CD. The centres used clinical protocols, in which healthcare workers were trained and which involved robust data collection. The Platform demonstrates the possibility of dramatically scaling up access and provides a robust model for national adoption in Bolivia. The 4D (Diagnose, Design, Deliver, Demonstrate) approach utilized in Colombia provides another example of a multi-stakeholder pilot project which delivers a comprehensive model of care [131]. This model is also based on a collaborative approach with government and stakeholders, and aims to deliver a model of care which is needs based and data driven, with associated health care system and health care worker capacity building to ensure sustainability of the project. The Precede-Proceed model upscaling prenatal screening in Guatemala has also demonstrated that a data driven comprehensive approach involving professionals from all aspects of care can improve diagnosis/treatment rates [132].

Future regional and multinational efforts to address the shortfall in diagnosis and treatment should build upon these local and regional efforts, with strong educational components for both physicians and public health officials that promote the cost-efficiency of such interventions while highlighting the moral imperative of addressing the needs of this generally indigent and marginalized patient population.

### Why do only some patients develop cardiac disease?

A critical knowledge gap exists as to why only 30% of infected individuals will progress to CCC [23]. Several host and parasite factors have been evaluated to understand this phenomenon. Thus far, the main factors identified as being related to cardiac damage are: Immune response to antigens of the parasite leading to fibrosing inflammation (T CD8 lymphocyte response), direct damage to myocytes by the presence of the parasite, damage of the neuronal cardiac system, autoantibodies against neuro-receptors, microvascular abnormalities, non-specific damage due presence of eosinophils and neutrophils, and oxidative stress. Moreover, different *T. cruzi* strains may be associated with different levels of cardiac toxicity [133,134]. While these factors likely act at least simultaneously, if not synergistically, there is growing evidence that parasite persistence is a necessary factor for disease progression.

A genetic susceptibility to develop CCC has been proposed, which may result from polymorphisms in genes related to the IFN-V axis that can lead to variations in the intensity of the immune response involved in the pathogenesis of the disease [135]. Further research is necessary to reveal the profile of patients who would benefit the most from trypanocidal treatment.

### Why are there poor cure rates in chronic patients?

Without a reliable measure of parasitological eradication, it is difficult to know true cure rates of chronically infected Chagas patients, hence serologic cure is a surrogate endpoint for treatment success. The reasons that there are much lower rates of sero-negativization in a treated infected adult relative to treated children remain unknown. Identification of biomarkers that more accurately measure efficacy could revolutionize clinical research and practice, and are a current subject of research initiatives [136]. Although potentially these would be improved indicators of ‘cure’, there are still surrogate endpoints in the absence of data that demonstrate a correlation with reduction in clinical event rates.

Age has been identified as an important predictor of cure; anti-parasitic treated children have higher rates and a prompter response to therapy than adults [98]. In addition, studies suggest a differential response to therapy depending on country of origin, suggesting variations in susceptibility to anti-parasitic agents depending on the parasite strain.

Host and parasite biology may also have additional implications for the response to anti-parasitic therapy. Research to date has also hypothesized that the quality of T-cell responses and immune-regulatory mechanisms might determine the pattern of cellular responses and the severity of disease in chronic *T. cruzi* infection. The quality of T-cell responses might be a key factor, not only in disease evolution but also in chemotherapy responsiveness [137]. This may also explain the differing response rate to anti-parasitic treatment among children in comparison with adults, as children have a T-cell profile associated with a more robust clinical response [138].

Additionally, animal model research demonstrates that a small proportion of trypomastigotes are dormant at any given time during infection, and are likely to be protected from anti-parasitic compounds in this state [139].

### Psychological aspects in comprehensive care of CD

“The patient with Chagas seeks something more than a tablet.” -Nilce Mendoza, an individual affected by CD residing in Spain [140]

Diagnosis of CD produces a range of reactions in individuals, from scepticism to fear and anxiety [141,142]. Those affected may feel understandable doubts about their diagnosis, given the lack of noticeable symptoms, and may not remember having been exposed to triatomines or other risk factors. Particularly in non-endemic areas, many newly diagnosed individuals have never heard of CD and encounter health care professionals who are similarly unaware of the disease and may be dismissive of its importance [143]. Often, individuals feel devastated by the knowledge that they have a potentially life-threatening disease, which can lead to depression, especially in those who suffer debilitating chronic symptoms [144]. For many, thoughts of CD have to be pushed to the background in light of a host of other social, economic, and emotional challenges involved in the daily struggle to survive. Further, affected individuals who have immigrated to non-endemic countries may be isolated from their traditional support networks and could find it difficult to engage with healthcare providers in host countries because of political, linguistic and cultural barriers [109]. Finally, health systems rarely offer support for the emotional and social challenges of living with CD, which are exemplified below in the words of an affected person:

“[…] I have family with Chagas. And of course, when they said I have Chagas, and at any moment I could die, of course I became sad. I thought a lot about the disease. I worried a lot.” [109]

### Patient and community involvement and empowerment in tackling CD

“It’s a fatal disease, and yet you don’t hear anything about it, it’s like a phantom disease that is killing people but nobody knows it exists, until they tell you, you have it. You always hear about diabetes, cancer, but [Chagas] disease is something that’s never heard anywhere, not even in the media.” -Sara, 60, El Salvador [109]

CD is largely a hidden disease, not only due to its long asymptomatic phase but also because it primarily (but not exclusively) affects politically and economically marginalized people [145]. This, combined with extremely low awareness of the disease among both providers and the general public, poses challenges for the organization and empowerment of people with CD. Nonetheless, patient organizations have a long history of being actively engaged in efforts to raise awareness of the disease, increase access to healthcare, and provide social and emotional support for affected people. For instance, the Pernambuco Association of Chagas and Heart Disease Patients in Brazil has worked since 1987, not only in educating community members but also providing social support to patients, operating in close collaboration with local healthcare services [146]. Patient organizations have emerged in endemic countries from Argentina to Mexico, and also in non-endemic countries. In 2010, the International Federation of Associations of People Affected by Chagas was formed and now consists of over 20 patient organizations. The road ahead for these patient organizations remains arduous, as the public profile of the disease, political commitment of governments, and access to political power of affected people all remain low.

### Improving access to resources for clinical decision support?

Clinical management resources and clinical guidelines have been written and updated in different regions and settings, but require further updating and a broader discussion of comprehensive CD care.

To date, three types of guidelines have been published:

1. Regional guidelines. The most recent regional clinical guideline is the one published by the Pan-American Health Organization/WHO (PAHO/WHO) [32]. This document focuses on evidence-based recommendations for the diagnosis and treatment of CD in adult and paediatric patients. Whom to screen, management of cardiomyopathy, and how to implement the recommendations are not addressed.
2. Endemic country national guidelines. Several Latin American countries have developed clinical guidelines for screening and treatment [147,148,149,150]. In general, these documents contain consistent guidelines and recommendations about prevention, epidemiological surveillance, vector control, and medical therapy. In particular, the Brazilian Ministry of Health has developed a very comprehensive Clinical Protocol (**Protocolo Clínico e Diretrizes Terapêuticas em Doença de Chagas**) [151], which includes a comprehensive consultation of key stakeholders and health professionals in order to help implement Brazil’s Consensus Guidelines [43].
3. Non-endemic country guidelines. Several guidelines, consensus documents, and review articles have been published during the last decade in non-endemic countries to address specific clinical topics [23,67,152,153,154], related to CD and catering to clinicians with no previous knowledge of the disease. However, these guidelines do not address whom to screen.

To address the need for a more comprehensive, up-to-date, and broadly applicable guideline document, the Chagas Coalition has formed a CD Guidelines Review Board. The aim of this committee is to address the gaps in prior guideline documents and to update existing recommendations based on new evidence. This document will use PAHO guidelines as a general framework, harmonizing when appropriate individual country guidelines to a general standard. The document also aims to be comprehensive in addressing all levels of CD care, incorporating recommendations from a diverse group of clinicians from a variety of specialties and communities.

## Roadblocks on the Roadmap to Chagas disease prevention, diagnosis and treatment

**Figure 12:**
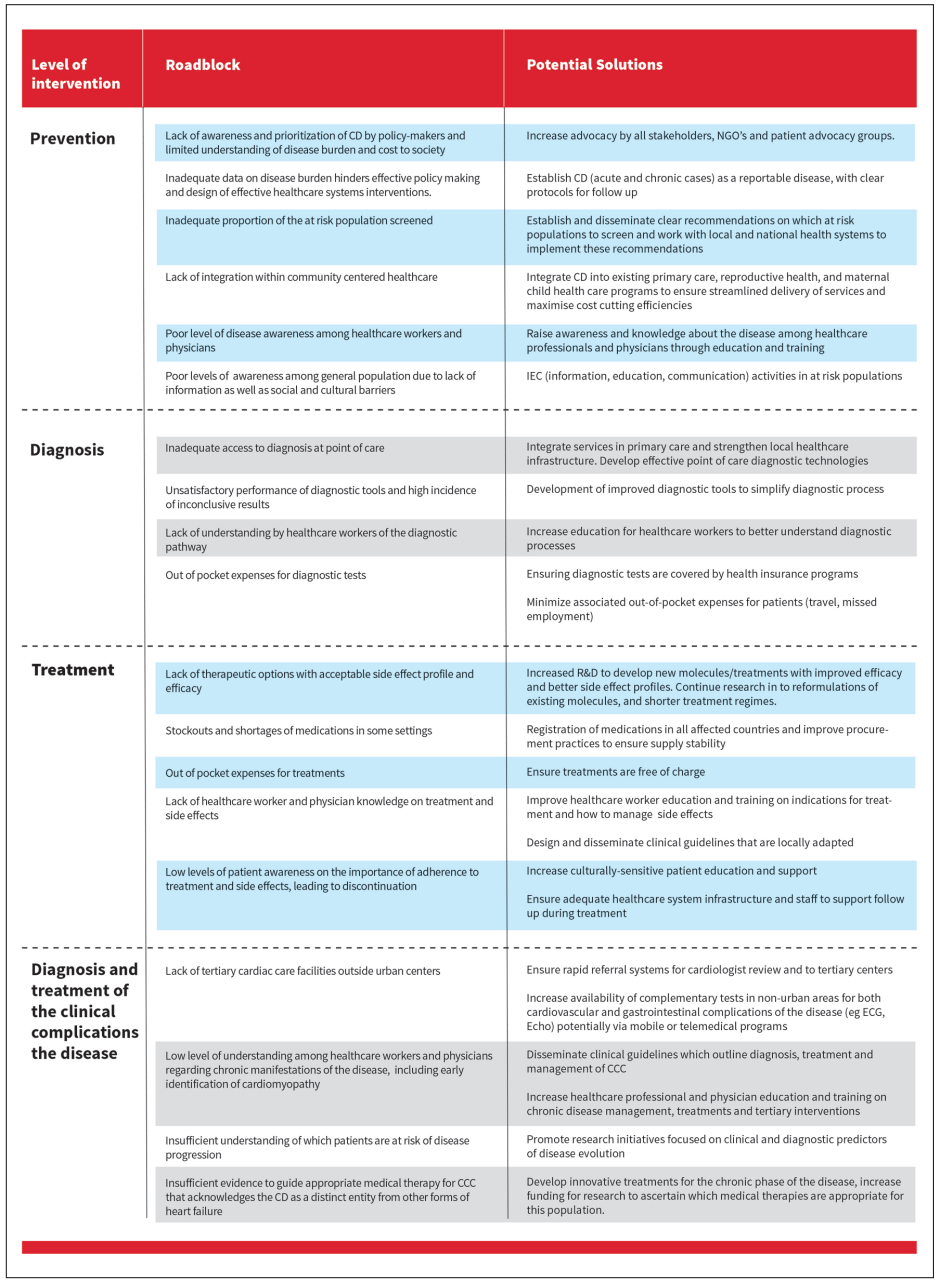
Roadblocks and proposed solutions at different levels of interventions.

Figure 12 overcoming the roadblocks to appropriate and timely prevention, diagnosis, and treatment for CD cannot be separated from the overarching goal of providing universal health coverage (UHC). Access to comprehensive, community-centred health services is the cornerstone of UHC and also to delivering appropriate care to individuals affected by CD. In addition, strategies to address the social determinants of health should underpin any comprehensive strategy on CD. At the policy level, there is a need for strengthening stewardship and governance to ensure that CD is embedded in government programmes and Ministry of Health policies. Financing policies should acknowledge that prevention and early diagnosis are cost-efficient programmes to reduce the burden of disease. Figure 12 summarizes the main roadblocks at four levels of intervention: prevention, diagnosis, treatment, and management of the clinical complications of the disease in its chronic stage.

Four factors common to all four steps on the pathway of care are discussed below: governance and advocacy, healthcare financing, the interaction between patients and caregivers, and information technology and registries.

### Factors influencing all four levels of care

#### Governance and advocacy

Since the term ‘Neglected Tropical Diseases’ was first coined in 2003, global advocacy actions addressing CD as one of this diverse group of diseases have been gaining momentum and are a vital component in addressing the unmet needs of patients with CD. As a disease surrounded by stigma and affecting marginalized populations, CD has been slow to gain the attention of governments and policy makers. Its inclusion within this group of neglected diseases, with an organized and concerted advocacy strategy, has meant an unprecedented rise in awareness over the last 10 years. The London Declaration of 2012 was the first global multi-stakeholder commitment on NTDs, bringing together civil society organisations, development agencies and private enterprise with an ambitious statement to control or eliminate 10 NTDs by 2020 [155]. This declaration ran in parallel to the second WHO 2020 Roadmap on NTDs, which is due to be updated in 2020 [156]. Chagas patient groups have also played an important role in recent years, by giving a voice to CD patients. Prompted by the International Federation of Associations of People Affected by Chagas (FINDECHAGAS), the 72nd World Health Assembly in Geneva marked the adoption of April 14th as World Chagas Day in 2019. The formation of the Chagas Coalition in 2012, which is a collaborative alliance of stakeholders working on the disease, has added cohesion to individual actors’ advocacy efforts and is also a valuable vehicle for pooling knowledge and technical expertise on CD.

Advocacy is a key component to enable the embedding of CD interventions and programmes in Ministries of Health and other public institutions. Such a structure favours the sustainability of health programmes and promotes better national coordination and financing. NGOs and other stakeholders also have an important role to play in encouraging this ‘buy in’ from governments. Where government participation is absent, advocacy is needed to raise CD in political agendas.

#### Financing

Fragmentation is a common characteristic of most Latin American healthcare systems, leading to inefficiencies in financing and increased expenditure. Healthcare budgets are also all experiencing increased strain due to aging populations, and the increasing prevalence of non-communicable diseases. While increasing healthcare spending on any individual disease may cause acute strain on healthcare budgets, it is clear that improving preventative efforts on CD as well as many other diseases, can effectively reduce this spending over time. In addition to these direct costs, CD has a substantial impact on worker productivity, by causing premature disability and death. It has been reported to represent a large economic burden [157], especially in endemic countries, with a global lifetime estimated burden of over $188 billion USD [10]. Therefore, financial investment in health care interventions to tackle CD should be framed in terms of the long-term savings to the economy as well as the healthcare system. Efforts to streamline expenditure that is already in place for CD, and maximising cross-cutting efficiencies by integrating interventions into existing health care infrastructure, are necessary and important measures to better use available funds.

#### Individuals affected by CD and their caregivers

CD is rarely discussed in mass media or health education campaigns, despite the fact affected people suffer stigma and even exclusion from jobs [145]. In addition to this, in non-endemic countries, a lack of documentation of Chagas patients may cause affected individuals to be reticent to draw attention to themselves or their medical needs. The marginalisation and stigmatisation of the disease exerts a significant toll, both practically in terms of access to care as well as psychologically.

Both patients and caregivers benefit from support in navigating the social and emotional impacts of the disease. This should ideally involve social workers and mental health professionals as well as community based support groups [146]. The geographic distance of facilities from patients’ communities can also be a major barrier in accessing care. Solutions such as providing transportation to referral centres or employing mobile clinics can help bridge this gap.

“Before, I couldn’t go [to the doctor], because I didn’t know how to drive. I had to wait for someone to take me; I depended on someone giving me a ride as a favour. And then I didn’t have money to pay for the appointment or the ride, or sometimes for lack of time, and I’ve had to neglect other tasks so I could go to the doctor.” -Renata, 36, Mexico [109]

Many of the populations most affected by CD are migrant populations. Given this characteristic, these groups may be indirectly or overtly excluded from healthcare systems in host countries and may face linguistic, political, and cultural barriers that hamper their efforts to access services and also to organize and advocate for the right to healthcare. Information about CD and its treatment options should be accessible to individuals affected by the disease and their caregivers, available in their preferred language, and written in a straightforward, culturally appropriate manner without unnecessary medical jargon.

#### Registries, information technology and digital health

Registration of patients with CD is the starting point not only for clinical follow-up and as a mechanism for improving treatment adherence, but also as the foundation for healthcare planning and population health initiatives, allowing for the allocation of resources and evaluating the impact of healthcare interventions over time. In some cases, national registries represent less than 1% of the total expected cases based on estimates of prevalence [71].

Up-to-date, interlinked information systems are also a key element of an integrated approach to patient care within the healthcare system, offering an important opportunity to make clinical information accessible to all health care professionals and health authorities involved in the pathway of care. Furthermore, such a system has the potential to eventually provide information that can be used by the health sector and related sectors to develop truly integrated approaches to health in all policies.

Health professionals working in remote and resource-limited places will benefit from the development of digital and web-based tools to access expert advice through teleconsultations on diagnostic tools [158], such as electrocardiogram [159] and echocardiogram [160]. Mobile health interventions, such as the use of text messaging, can also be useful for both health professionals in the care of CD patients and for improving the adherence of patients to clinical care [161].

Platforms for e-learning are also a valuable tool to expand access to medical education, online access to guidelines and medical updates.

In the future, cognitive computing combined with artificial intelligence offers the prospect of readily accessible tools for patient self-assessment, including symptom status and side effects of therapy. Such tools may also be beneficial in monitoring adherence to treatment and improving patient health literacy and awareness of the disease [162].

## Conclusions

CD is a complex but entirely preventable and treatable disease. The barriers affecting access to diagnosis, treatment and care are complex, and a strategic and comprehensive approach is required to address these roadblocks in the various settings where they exist.

This Roadmap provides an example of an ideal patient care pathway for CD (Figure 6), and explores the roadblocks along this pathway, considering potential solutions based on available research and evidence in practice. To move from prescribed global recommendations to local and national implementation, a number of specific actions are required to plan, design, and implement change.

The challenge remains of how to move from recommendations to practice implementation, and in that sense, it is crucial to adapt these recommendations to each particular framework at the national level. This means considering the particularities of the healthcare system and policy environment, and identifying specific barriers and potential strategies on a regional, national and local level.

**Figure 13:**
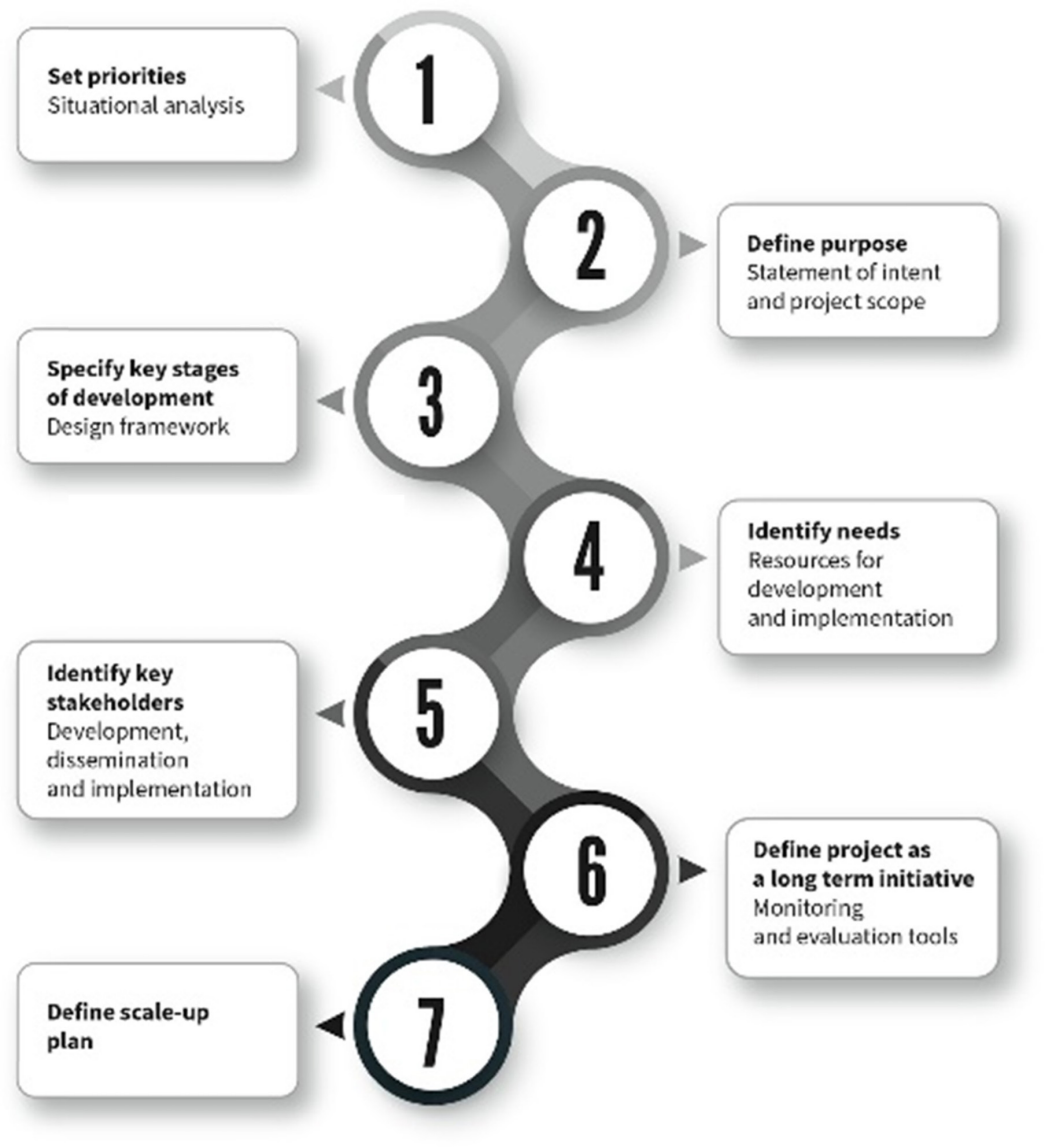
WHF Implementation framework.

The WHF implementation framework in Figure 13 offers a step-by-step approach to specific action areas and highlights the importance of an integrated approach across multiple care settings. Moving from a global roadmap initiative to a national call for action requires involvement and concerted action from national ministries of health, health care system decision-makers, and health care professionals. Patients are a pivotal part of these efforts, with the support of their families and caregivers, and civil society has an important role to play as well.

Bringing key leaders and stakeholders together for national roundtable discussions to consider a unified CD agenda based on national and global needs should be considered a necessary first step.
